# First person – Diego Baronio

**DOI:** 10.1242/dmm.049419

**Published:** 2022-02-23

## Abstract

First Person is a series of interviews with the first authors of a selection of papers published in Disease Models & Mechanisms, helping early-career researchers promote themselves alongside their papers. Diego Baronio is first author on ‘ Abnormal brain development of monoamine oxidase mutant zebrafish and impaired social interaction of heterozygous fish’, published in DMM. Diego is a PhD student in the lab of Pertti Panula at the University of Helsinki, Helsinki, Finland, investigating mechanisms involved in the pathophysiology of neurodevelopmental disorders.


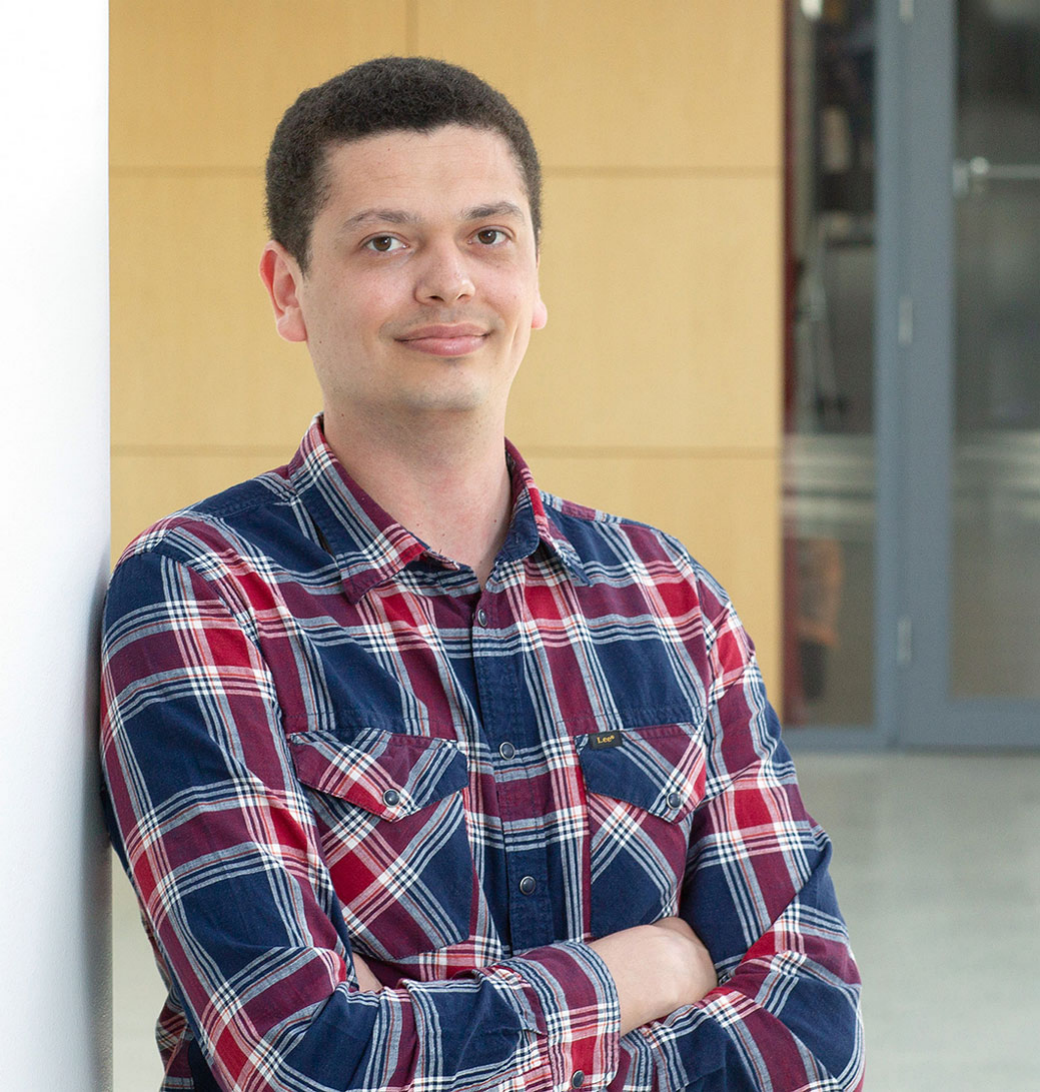


**Diego Baronio**

**How would you explain the main findings of your paper to non-scientific family and friends?**

Monoamine oxidases (MAOA and MAOB) are enzymes responsible for the metabolism of neurotransmitters called monoamines. Low activity of MAOA has been detected in patients with autism spectrum disorder (ASD). This may lead to increased levels of monoaminergic neurotransmitters, such as serotonin, which have a critical role in brain development. We decided to use zebrafish to study the behavioural and developmental consequences of MAO deficiency and to verify if this model organism would be a suitable tool to study monoaminergic-associated neurodevelopmental disorders, such as ASD. Zebrafish lacking Mao (*mao*^−/−^) showed altered expression of genes that are important for brain development, disrupted monoaminergic systems and early death. Heterozygous animals (*mao*^+/−^), which had a 50% reduction in the expression of *mao* and decreased Mao activity, presented impaired social behaviour. Our findings indicate that these are promising tools to study the roles of MAO and monoamines during brain development and the behavioural outcomes of MAO deficiency.

**What are the potential implications of these results for your field of research?**

Alterations in monoaminergic systems can impair brain functions and lead to mental illness. *mao*^−/−^ zebrafish are a promising tool to understand how MAO and monoaminergic systems affect different aspects of brain development. Additionally, they could be used in high-throughput screening and drug testing, allowing the identification of new compounds that could prevent or restore monoaminergic alterations. *mao*^+/−^ zebrafish presented only a mild impairment in social behaviour. However, since environmental and genetic components are known to play a role on the underlying mechanisms of ASD, *mao*^+/−^ fish could be used in future studies assessing developmental and behavioural outcomes of interaction between MAO genotype and environmental factors.

“The zebrafish is an attractive tool in neuroscience research because it shares relevant neurochemical aspects with humans.”

**What are the main advantages and drawbacks of the model system you have used as it relates to the disease you are investigating?**

The zebrafish is an attractive tool in neuroscience research because it shares relevant neurochemical aspects with humans. Neurotransmitters systems components, such as transporters, receptors and enzymes, can be easily studied and visualized in the zebrafish brain through different imaging techniques at an early stage of development. Additionally, the wide range of behaviours displayed by zebrafish also contribute to its appreciation as a model organism for brain disorders. However, unlike mammals, zebrafish possess only one form of Mao, which resembles mammalian MAOA and MAOB but also shows distinct characteristics that differ from both mammalian enzymes. Therefore, the structure, activity and function of the zebrafish Mao should be taken into consideration when using this model system.

**Figure:**
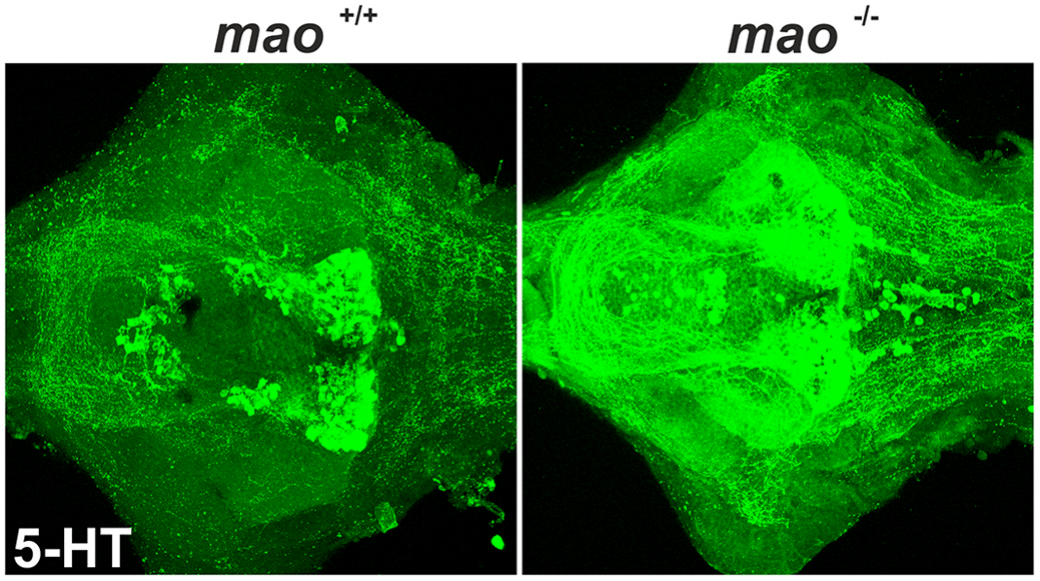
Increased serotonin immunoreactivity in the brain of a zebrafish lacking monoamine oxidase in comparison with the brain of a wild-type sibling.

**What has surprised you the most while conducting your research?**

I was surprised with the possibilities zebrafish offer as a model organism. The genetic tools that can be used to generate mutants and the rich repertoire of behaviours fascinated me. It was fun to come up with new behavioural paradigms and to build the necessary apparatuses to test the animals.

**Describe what you think is the most significant challenge impacting your research at this time and how will this be addressed over the next 10 years?**

It is necessary to provide novel insights into the neurobiological mechanisms of ASD. Once we understand more about these mechanisms, we will be able to develop new drugs to treat the core symptoms of this disorder. There are different animal models, generated by various genetic and pharmacological interventions, which show phenotypic heterogeneity that resembles the variety of clinical manifestations observed in ASD patients. These models are important tools to tackle the challenges in ASD research.

[…] a supervisor's job is not limited to discussing experiments and what directions to take in a research project.

**What changes do you think could improve the professional lives of early-career scientists?**

A career in science can be unstable because of the lack of funding and reduced number of permanent positions. In my opinion, a supervisor's job is not limited to discussing experiments and what directions to take in a research project. It is very important that they are willing to discuss with early-career scientists about career pathways and to share their experiences on how they succeeded in academia. I was lucky to receive all the support necessary from my supervisor to develop my research project and to plan my future in science.

**What's next for you?**

The next steps for me are to finish my PhD thesis and to continue my career as a postdoctoral researcher. I would like to continue studying ASD and dissect neural circuit mechanisms associated with behavioural impairments that are characteristic of this disorder.
